# The Reporting on ERAS Compliance, Outcomes, and Elements Research (RECOvER) Checklist: A Joint Statement by the ERAS^®^ and ERAS^®^ USA Societies

**DOI:** 10.1007/s00268-018-4753-0

**Published:** 2018-08-16

**Authors:** Kevin M. Elias, Alexander B. Stone, Katharine McGinigle, Jo’An I. Tankou, Michael J. Scott, William J. Fawcett, Nicolas Demartines, Dileep N. Lobo, Olle Ljungqvist, Richard D. Urman, the ERAS® Society and ERAS® USA, Kevin Elias, Maria Adamian, Frederic Bushnell, Arman Dagal, Chad Engan, Joseph Marcotte, Katharine McGinigle, Nicollete Pauksta, Venu Pillarisetty, Jo’An Tankou

**Affiliations:** 1 Division of Gynecologic Oncology, Department of Obstetrics, Gynecology and Reproductive Biology, Brigham and Women’s Hospital, Harvard Medical School, 75 Francis Street, Boston, MA 02115 USA; 2 Department of Anesthesiology, Perioperative and Pain Medicine, Brigham and Women’s Hospital, Harvard Medical School, Boston, MA USA; 3 Division of Vascular Surgery, University of North Carolina School of Medicine, Chapel Hill, NC USA; 4 Department of Anesthesiology, Virginia Commonwealth University Health System, Richmond, VA USA; 5 Department of Anesthesiology, Perelman School of Medicine, University of Pennsylvania, Philadelphia, PA USA; 6 Department of Anaesthesia, Royal Surrey County Hospital and University of Surrey, Guilford, UK; 7 Department of Visceral Surgery, Lausanne University Hospital CHUV, Lausanne, Switzerland; 8 Gastrointestinal Surgery, Nottingham Digestive Diseases Centre and National Institute for Health Research (NIHR) Nottingham Biomedical Research Centre, Nottingham University Hospitals NHS Trust and University of Nottingham, Queen’s Medical Centre, Nottingham, NG7 2UH UK; 9 Department of Surgery, Faculty of Medicine and Health, School of Health and Medical Sciences, Örebro University, Örebro, Sweden

## Abstract

**Background:**

Enhanced recovery after surgery (ERAS) programs are multimodal care pathways designed to minimize the physiological and psychological impact of surgery for patients. Increased compliance with ERAS guidelines is associated with improved patient outcomes across surgical types. As ERAS programs have proliferated, an unintentional effect has been significant variation in how ERAS-related studies are reported in the literature.

**Methods:**

To improve the quality of ERAS reporting, ERAS^®^ USA and the ERAS^®^ Society launched an effort to create an instrument to assist authors in manuscript preparation. Criteria to include were selected by a combination of literature review and expert opinion. The final checklist was refined by group consensus.

**Results:**

The Societies present the Reporting on ERAS Compliance, Outcomes, and Elements Research (RECOvER) Checklist. The tool contains 20 items including best practices for reporting clinical pathways, compliance auditing, and formatting guidelines.

**Conclusions:**

The RECOvER Checklist is intended to provide a standardized framework for the reporting of ERAS-related studies. The checklist can also assist reviewers in evaluating the quality of ERAS-related manuscripts. Authors are encouraged to include the RECOvER Checklist when submitting ERAS-related studies to peer-reviewed journals.

## Introduction

Enhanced recovery after surgery (ERAS) programs are multimodal care pathways designed to minimize the physiological and psychological impact of surgery for patients. ERAS pathways rely on multidisciplinary teams and require coordinated interventions in all parts of perioperative care, from the initial preoperative consultation through the hospitalization and onward to the return of the patient to normal activities of daily living [1]. ERAS programs reduce hospital lengths of stay and postoperative complications while decreasing the costs of care for patients and health systems [2, 3]. Most ERAS pathways are designed around approximately 25 core elements outlined by the ERAS^®^ Society [4]. There are data to suggest that increased compliance with these core elements is associated with improved outcomes across surgical types [5–8].

In recent years, research in ERAS has expanded significantly. ERAS programs have expanded beyond colorectal surgery to other surgical disciplines and have been implemented successfully in pancreatic surgery, thoracic surgery, liver resection, urologic surgery, gynecologic surgery, and emergency surgery, among others [5, 9–13]. An unintentional effect of this rapid expansion has been significant variations in how ERAS studies are reported [14]. The COMPAC (Core Outcome Measures in Perioperative and Anaesthetic Care) group has embarked on an effort to standardize the outcomes reported in perioperative medicine https://www.niaa-hsrc.org.uk/HSRC-COMPAC. While there are some efforts to apply a similarly tiered approach to reporting ERAS outcomes, we believe that a truly comprehensive ERAS report should detail not only the outcomes, but also the process by which those outcomes were achieved [15]. Like any other scientific enterprise, the methods should contain sufficient detail to enable another group to reproduce the results. Moreover, as ERAS protocols have now been in place at many sites for years, there is a need to mature ERAS studies beyond the common retrospective comparisons to pre-ERAS historic controls. These lower-quality studies tend to magnify the benefits of the intervention being studied. Indeed, properly designed prospective studies have been revealing, showing that interventions which may improve outcomes under traditional perioperative management do not necessarily confer additional benefit when both groups are on ERAS pathways [16]. Hence, there is a need to formalize ERAS-related research such that meaningful results are reproducible and generalizable. We created the Reporting on ERAS Compliance, Outcomes, and Elements Research (RECOvER) Checklist to provide authors and reviewers with a set of standards for excellence in reporting ERAS-related studies. This checklist is not a guideline itself—in fact, quite the opposite. It is a tool to assist authors when reporting outcomes on guidelines already in practice and, if anything, should prompt reviewers or readers to ask why elements in a report are not described or may not be appropriate. Our goal is to encourage reproducibility in clinical studies, acknowledging the different variables which may influence ERAS outcomes and the different ways in which ERAS has been implemented in units around the world. This is not a meta-analysis; rather, we aim to improve study reporting so that future meta-analyses can be more easily performed by ensuring sufficient information on ERAS practice and description of outcomes.

## RECOvER development

A checklist and statement were developed by a small working group of volunteers from ERAS^®^ USA (the American chapter of the ERAS^®^ Society). A subcommittee (KME, KM, JIT) from the ERAS^®^ USA Research Committee reviewed ERAS-related publications from across different medical specialties and study designs. In developing the checklist, subcommittee members were asked to review 10–15 manuscripts each from anesthesia, surgery, or general interest journals and tasked to define best practices in ERAS reporting. Questions to be addressed were:

1. How are the study groups defined?
2. How do the authors convey the implementation of ERAS principles?
3. What steps are taken to assess compliance?
4. What outcomes are measured?

This subcommittee developed an initial list of 32 items for inclusion in a checklist of best practices. After discussion with the larger committee, this number was reduced to 20 items to focus the checklist on elements related to ERAS rather than to general guidelines for best practices in research reporting. The total number of elements was reduced by removing those redundant with general reporting guidelines, for example the Enhancing QUAlity and Transparency Of health Research (EQUATOR) network guidelines, or by combining similar elements to make the checklist more concise [17]. Reconciliation in the rare cases where disagreement occurred was achieved by following the majority opinion of the authors. After agreement in the Research Committee was achieved, the checklist was circulated to members of the ERAS^®^ Society Executive Committee (MJS, WJF, ND, DNL, and OLP) for further comments. Following feedback from members of the society, the final number of 20 items was confirmed by consensus agreement by the members of the Research Committee and Standards and Protocols Committee of ERAS^®^ USA (ABS, RDU).

## RECOvER items

The complete list of checklist items is shown in Table 1. We recommend that authors publishing research in the field of ERAS include this checklist with their submissions and indicate the location of each item in the manuscript. This is a framework for guidance. It should facilitate publication rather than serve as a barrier. The ultimate decision to accept or reject manuscripts is with the individual journal editors; the guidance is not proscriptive. Each item in the checklist pertains to a particular point in the course of conducting an ERAS research study, from conceptualization to data analysis and the writing of the manuscript. Therefore, we recommend that the researchers consult the checklist as early as possible during the study planning process. Below we provide a detailed description of each checklist item, followed by some examples.

**Table 1:** RECOvER Checklist for reporting of enhanced recovery research

	Item	Recommendation	Page
*Title*			
Title	1	Indicate that this is an enhanced recovery study in the title	
*Introduction*			
Background	2	Explain the area of uncertainty that the study seeks to address	
Guidelines	3	If a published set of enhanced recovery guidelines exists for this procedure, include a reference to the guidelines	
Outcomes	4	Define the primary outcome and any key prespecified secondary outcomes for the study	
*Methods*			
IRB approval	5	Give the Institutional Review Board/Ethics Committee name and approval number. If permission was not required, reasons should be stated	
Study design	6	Indicate what type of study is presented (randomized controlled trial, cohort, cross-sectional, etc.) The individual guidelines for the type of study should be followed (e.g., CONSORT for randomized controlled trial, STROBE for cohort studies, etc.)	
Setting	7	Describe whether this is a single or multicenter study, the type of practice (academic vs. community, tertiary vs. primary), and the providers (limited group or all providers on a service)	
Timing	8	Describe periods of recruitment, time points at which outcomes assessed, and follow-up	
Participants	9	Define study inclusion and exclusion criteria	
Enhanced recovery protocol	10	Describe when the enhanced recovery protocol was implemented relative to the study period	
	11	Provide a flow diagram or table through the continuum of care detailing the enhanced recovery protocol including the following elements:	
		(a) Preadmission patient education regarding the protocol	
		(b) Preadmission screening and optimization as indicated for nutritional deficiency, frailty, anemia, HbA1c, tobacco cessation, and ethanol use	
		(c) Fasting and carbohydrate loading guidelines	
		(d) Preemptive analgesia (dose, route, timing)	
		(e) Anti-emetic prophylaxis (dose, route, timing)	
		(f) Intraoperative fluid management strategy	
		(g) Types, doses, and routes of anesthetics administered	
		(h) Patient warming strategy	
		(i) Management of postoperative fluids	
		(j) Postoperative analgesia and anti-emetic plans	
		(k) Plan for opioid minimization	
		(l) Drain and line management	
		(m) Early mobilization strategy	
		(n) Postoperative diet and bowel regimen management	
		(o) Criteria for discharge	
		(p) Tracking of post-discharge outcomes	
Enhanced recovery auditing	12	Describe the audit system for compliance with the enhanced recovery protocol and how compliance data are measured	
Outcomes	13	(a) Explain the criteria for assessing primary and secondary outcomes	
		(b) Distinguish among clinical, functional, administrative, and quality of life outcome measures	
PROs	14	If patient questionnaires are used, provide references to validation of these study instruments	
*Results*			
Patient population	15	Use a flow diagram to explain the derivation of the study population	
		(a) Provide a Table I with the key demographic and clinical features of the study population	
		(b) Indicate number of participants with missing data for each variable of interest	
Enhanced recovery compliance	16	Provide a Table II with average compliance for each enhanced recovery protocol element and present a comparison of the variation in enhanced recovery compliance among the study groups	
Correlations	17	Perform logistic regression to correlate the change in primary outcome with the study intervention	
*Discussion*			
Context	18	Explain what the study adds to the body of knowledge regarding the study intervention within the context of enhanced recovery after surgery care	
Limitations	19	Discuss the limitations of the study and how these might temper the findings	
*Other information*			
Funding	20	Document all sources of funding and potential conflicts of interest for the study authors	

*RECOvER* Reporting on ERAS Compliance, Outcomes, and Elements Research, *CONSORT* Consolidated Standards Of Reporting Trials, *STROBE* STrengthening the Reporting of OBservational studies in Epidemiology, *PROs* patient-reported outcomes

Reporting standards begin with the title page. ERAS studies should refer to enhanced recovery within the title (item 1), which will facilitate queries for future systematic reviews. The title should also relay the study type and surgical procedure studied—for example, a retrospective cohort study of patients undergoing robotic-assisted pancreaticoduodenectomies. In the introduction, the authors should explain the specific area of clinical uncertainty being addressed within the context of ERAS (item 2)—for instance, whether high-volume or high-concentration local anesthetic provides superior local analgesia to the incision. As the ERAS^®^ Society and other perioperative research societies have published guidelines for many procedures, existing guidelines, if applicable, should be referenced (item 3). The primary outcome for the study should be clearly stated in the introduction, as well as key secondary outcomes of interest (item 4). While many ERAS studies have focused on administrative outcomes, such as hospital length of stay, or clinical outcomes, such as wound infection or transfusion rates, there is considerable need for more ERAS studies addressing functional outcomes. The latter might include outcomes such as return to work or discharge to home rather than to a rehabilitation facility [18–20]. There is also a need for more studies examining non-surgical perioperative morbidity within the context of established ERAS programs, such as the consequences of preoperative anxiety or postoperative delirium [21, 22].

Within the materials and methods, all ERAS studies should describe the Institutional Review Board (IRB) or Ethics Committee review or explain the rationale for IRB/Ethics Committee exemption (item 5). The study design, including whether this is a prospective or retrospective study, should be evident (item 6). The design description should include details on the clinical context, including the setting (item 7), timing (item 8), and selection of patients (item 9) for the study. This includes the type of hospital, period of recruitment, and inclusion and exclusion criteria for the study. The authors should place the report temporally with respect to the introduction of ERAS at the institution (item 10), including differentiating pre-ERAS from post-ERAS groups of patients. An explicit statement regarding the dates of introduction of ERAS at the institution, if possible, is preferred. Paramount to ERAS-related studies is documentation that the principles of enhanced recovery are being followed (item 11). While the literature is rife with reports of ERAS failures, a closer inspection may reveal a lack of compliance with ERAS concepts [23, 24]. A detailed description of the ERAS pathway should cover all phases of care (preadmission, preoperative, intraoperative, post-anesthesia care, inpatient, post-discharge, and follow-up care). The description should also include the management strategies for perioperative optimization, opioid-sparing analgesia, fluid management, avoidance of starvation, nutritional care, mobilization, and discharge. These elements must then be related to an audit system for pathway compliance (item 12), whether the ERAS^®^ Interactive Audit System^®^ (EIAS^®^) or local databases. The report should include a list of the metrics that enter into the compliance calculation. Similarly, outcomes, both primary and secondary, should be clearly defined a priori (item 13), and whenever patient-reported outcomes (PROs) or surveys are introduced, these should use validated and referenced instruments (item 14).

Results reporting in ERAS should reflect similar transparency to the methods. The reader should be able to visualize the derivation and composition of the study population (item 15). A description of the population should identify what proportion of all patients undergoing the procedure of interest is being reported and the reasons for exclusion from the study. The outcomes results should be displayed in the context of the actual compliance with the ERAS elements (item 16). Again, this requires an auditing system in place so that percentage compliance with the protocol can be plotted against the outcomes of interest. Whenever possible, regression analysis techniques should be used to test for independent associations between the primary outcome and the intervention under study (item 17). For example, in a study of ambulation from the postoperative recovery unit compared to ambulation on reaching the inpatient ward, where the primary outcome is actually the 6-minute walk test result at 2 weeks after surgery, a regression analysis should include confounders for early ambulation such as time of day, neuraxial analgesia, or receipt of opioids.

The discussion of ERAS studies should place the work within the larger context of ERAS-related care (item 18). Authors should strive to link the findings with tangible opportunities to improve clinical practice. A study that shows a decrease in visual analog scale (VAS) pain scores from 5 to 4 is of much less impact on the field than a similar study that examines the proportion of patients discharged to home rather than to a rehabilitation facility. Authors sometimes consciously or subconsciously overinterpret the results of their study, that is, they add “spin” to the conclusions of a scientific report. Spin is defined as a non-neutral way of reporting that distorts the interpretation of results and misleads readers [25]. An appraisal of the study limitations should be candid (item 19), including critiques of the ERAS protocol itself, if indicated. Finally, authors must be open regarding funding support and possible conflicts of interest (item 20).

## RECOvER scope

An example of the RECOvER Checklist appears in Table 2. The primary aim of the RECOvER Checklist is to ensure an ERAS-specific addendum accompanies ERAS-related studies so that the reader can assess the ERAS-specific elements. The checklist is not mandatory; rather, it serves as a framework to make it easier to compare ERAS studies and assemble future systematic reviews and meta-analyses.

**Table 2:** Example of a RECOvER Checklist

	Item	Recommendation	Page
*Title*			
Title	1	Gum chewing improves recovery of gut function within an enhanced recovery protocol for hepatic resection	1
*Introduction*			
Background	2	Whether gum chewing offers additional benefit for functional gut recovery after liver resection beyond other enhanced recovery elements is uncertain	3
Guidelines	3	Melloul E, et al. World J Surg 2016 Oct;40(10):2425–2440	3
Outcomes	4	*Primary outcome* Time to first bowel movement after surgery *Secondary outcomes* Incidence of postoperative ileus, length of stay, incidence of postoperative emesis	3
*Methods*			
IRB approval	5	General Hospital IRB #123456	4
Study design	6	Retrospective cohort study	4
Setting	7	Single institution, community-based academic hospital with stable group of surgeons during the study period	5
Timing	8	Patients included from March 2013–May 2015, events assessed daily from surgery to discharge, all patients followed until 2-week postoperative visit	5
Participants	9	*Inclusion criteria* 18+ years old, participating in the enhanced recovery protocol, undergoing hepatic resection, not admitted to ICU postoperatively *Exclusion criteria* Age <18, unable or unwilling to participate in enhanced recovery protocol, other surgical procedures, ICU admission	5
Enhanced recovery protocol	10	enhanced recovery protocol was initiated in March 2012	6
	11	Provide a flow diagram or table through the continuum of care detailing the enhanced recovery protocol including the following elements:	7
		(a) Preadmission patient education regarding the protocol All patients receive an informational packet, watch a 10-minute video, and attend a 1-h preoperative educational class	
		(b) Preadmission screening and optimization for nutritional deficiency, frailty, tobacco cessation, and ethanol use Patients are screened for nutritional deficiency using the NRS scoring system, frailty using the scoring model published by Kim et al. and referred preoperatively for tobacco and ethanol counseling	
		(c) Fasting and carbohydrate loading guidelines Normal diet until midnight, clear liquids until 2 h before surgery, 300-ml isotonic beverage containing a total of 50 grams of maltodextrin finished 2 h before surgery	
		(d) Preemptive analgesia (dose, route, timing) 300 mg celecoxib, 500 mg acetaminophen both oral given in pre-op	
		(e) Anti-emetic prophylaxis (dose, route, timing) 4 mg ondansetron and 8 mg dexamethasone given intravenously prior to emergence	
		(f) Intraoperative fluid management strategy Esophageal Doppler monitoring of stroke volume variation	
		(g) Types, doses, and routes of anesthetics administered Continuous propofol, intravenous lidocaine, and low-dose ketamine infusion, no volatile anesthesia	
		(h) Patient warming strategy Forced warm air and intravenous fluid warmer	
		(i) Management of postoperative fluids 0.5 ml/kg/h × 6 h	
		(j) Postoperative analgesia and anti-emetic plans 0.25% liposomal bupivacaine wound infiltration, 500 mg acetaminophen and 600 mg ibuprofen every 6 h orally, 4 mg ondansetron every 6 h intravenously as needed	
		(k) Plan for opioid minimization First-line analgesic 25 mg tramadol every 6 h orally as needed, increased to 50 mg tramadol if needed, followed by addition of IV lidocaine infusion if needed, followed by pregabalin 100–300 mg every 8 h if needed, followed by 5–10 mg oral oxycodone for breakthrough pain	
		(l) Drain and line management No routine wound drains, Foley catheter removed in OR	
		(m) Early mobilization strategy Patients ambulate to chair in PACU, ambulate × 3 starting postoperative day 0, out of bed all meals, out of bed 8 h per day starting postoperative day 1	
		(n) Postoperative diet and bowel regimen management Clear liquids post-op day 0, regular diet beginning post-op day 1, standing MiraLax daily beginning post-op day 0	
		(o) Criteria for discharge Tolerating at least 2000 ml po daily, voiding independently, pain well controlled on oral medication, ambulating in hallways	
		(p) Tracking of post-discharge outcomes Patients contacted by office through daily email survey	
Enhanced recovery auditing	12	All enhanced recovery elements charted by physician assistant into Enhanced Recovery Interactive Audit System (EIAS)	8
Outcomes	13	(a) *Primary outcome* Bowel movement as documented by RN *Secondary outcomes* Per patient report as collected by physician assistant interview	9
		(b) Clinical outcomes	
PROs	14	European Organisation for Research and Treatment of Cancer Quality of Life Questionnaire-C30 (J Clin Epidemiol 2014)	9
*Results*			
Patient population	15	See Figure 1 (or similar)	10
		(a) See Table 1 (or similar)	11
		(b) Participants with missing data indicated in Table 1 footnotes	11
Enhanced recovery compliance	16	Table II provides enhanced recovery compliance for the gum-chewing versus non-gum-chewing groups for 15 metrics from the enhanced recovery pathway	12
Correlations	17	Table III provides logistic regression examining gum chewing with respect to primary and secondary outcomes	13
*Discussion*			
Context	18	Study suggests that gum chewing has additional benefits to standard bowel regimen, early feeding, and laxative guidelines for promoting early return of gut function	15
Limitations	19	Not a prospective study, did not have sufficient power to subdivide patients by indication for hepatic resection, poor compliance among the cohort with respect to early mobilization and termination of intravenous fluids	16
*Other information*			
Funding	20	Support from departmental grant	2

*RECOvER* Reporting on ERAS Compliance, Outcomes, and Elements Research, *IRB* Institutional Review Board, *ICU* intensive care unit, *NRS* nutrition risk screening, *PACU* post-anesthesia care unit

## RECOvER Checklist availability

The RECOvER Checklist will be made available on the ERAS^®^ USA as well as the ERAS^®^ Society websites. It is free and available as an open access document to support higher-quality and more consistent reporting of ERAS research.
